# Use of interfering RNA to investigate the role of endogenous gastrin in the survival of gastrointestinal cancer cells

**DOI:** 10.1038/sj.bjc.6603588

**Published:** 2007-01-30

**Authors:** A M Grabowska, J Hughes, S A Watson

**Affiliations:** 1 Academic Unit of Cancer Studies, D Floor, West Block, Queen's Medical Centre, University Hospital, Nottingham NG7 2UH, UK

**Keywords:** gastrin, gastrointestinal cancer, interfering RNA

## Abstract

Gastrin isoforms, acting through a variety of receptors, have proliferative and anti-apoptotic effects on gastrointestinal (GI) cancers. A small interfering RNA (siRNA) targeting the gastrin gene was used to investigate the role of endogenous gastrin in GI cancer cell survival. Downregulation of the gastrin gene in siRNA-transfected cells was measured using real-time reverse transcriptase–PCR. The most effective siRNA was tested in a panel of GI cancer cell lines at various concentrations and time points, and the effect on cell survival and apoptosis was measured using methyl thiazoyl tetrazolium (MTT) and caspase 3 activation assays. Gastrin siRNA reduced gene expression by more than 90% in a range of GI cancer cell lines. Downregulation of the gastrin gene was dose-dependent and effective over approximately 1 week *in vitro*. However, downregulation at the protein level was delayed by 3–4 days. Gastrin siRNA-transfected cells showed up to a 60% reduction in growth and up to a 50% increase in apoptosis compared with control siRNA-transfected cells. The effects were most marked in the cell line with the highest constitutive level of gastrin gene expression (human metastatic colon, C170HM2) and in epidermal growth factor (EGF)-treated cells as the gastrin promoter contains an EGF-response element, gERE. The ability of the siRNAs to reduce survival of these GI cell lines is further evidence of the importance of autocrine and/or intracrine gastrin loops in GI cancer, where expression of the gastrin gene and autonomous gastrin appears widespread.

Gastrin is produced by G cells in the antral mucosa, and in addition to its established role in gastric acid secretion, it plays a major role in tumorigenesis in the gastrointestinal (GI) tract. Gastrin expression is frequently increased in colonic, pancreatic and gastric adenocarcinomas compared with normal mucosa (Finley *et al*, 1993; Goetze *et al*, 2000; Henwood *et al*, 2001). It is expressed early in the development of GI adenocarcinomas (Smith and Watson, 2000; Henwood *et al*, 2001), and in an APC^*Min*^ mouse model, hypergastrinaemia promoted the progression of premalignant lesions (Watson and Smith, 2001). Tumour growth factor-alpha and epidermal growth factor (EGF) are involved in upregulation of gastrin expression in a number of colon cancer cell lines through binding to an established EGF response element, gERE, within the gastrin promoter (Howell *et al*, 1995; Watson *et al*, 2000).

In G cells, gastrin is processed to a 17-amino-acid amidated active product. However, in GI epithelial cells, processing is incomplete, resulting in secretion of the precursor peptide, progastrin, and a range of intermediate products, of which the most well-characterised is gly-gastrin (Rehfeld *et al*, 2004). Whereas amidated gastrin acts through the CCK-2 receptor (CCK-2R) (Shulkes and Baldwin, 1997), progastrin and gly-gastrin bind with low affinity to this receptor (Baldwin *et al*, 2001; Dockray *et al*, 2001). Furthermore, the biological effects of progastrin and gly-gastrin are not blocked by CCK-B receptor antagonists (Seva *et al*, 1994; Stepan *et al*, 1999; Baldwin *et al*, 2001), and amidated gastrin and gly-gastrin stimulate different downstream signalling events (Todisco *et al*, 2001). Potential alternative receptors for progastrin and gly-gastrin have been suggested (Seva *et al*, 1994; Baldwin, 1995a; Singh *et al*, 1995; Smith *et al*, 2002; Ahmed *et al*, 2004).

Exogenously applied gastrin and its precursors have a range of downstream effects on GI cancer cells. Amidated gastrin, gly-gastrin and progastrin promote growth of a range of cell lines *in vitro* and *in vivo* (Watson *et al*, 1989; Seva *et al*, 1994; Baldwin, 1995b; Stepan *et al*, 1999; Baldwin *et al*, 2001) and upregulate expression of a range of anti-apoptotic proteins, including Akt and bcl-2 (Konturek *et al*, 2003; Harris *et al*, 2004a; Ramamoorthy *et al*, 2004). Gly-gastrin promotes invasion of human colon cancer cells by upregulating expression of matrix metalloproteinases (Baba *et al*, 2004), by increasing the rate of metastasis formation *in vivo* (Kermorgant and Lehy, 2001) and by promoting blood vessel formation through the stimulation of endothelial cells (Clarke *et al*, 2006).

The role of endogenously produced gastrin has been investigated through the use of antibodies to gastrin and CCK-2R antagonists (Hoosein *et al*, 1988; Smith *et al*, 1996). However, such reagents may underestimate the role of gastrin, as a cocktail of reagents would be needed to inhibit all biologically active products of the gastrin gene and may be ineffective against gastrin-derived peptides that have not yet been identified.

This study uses small interfering RNAs (siRNAs) to reduce gastrin expression in a range of GI cancer cells in order to investigate the role of endogenously produced gastrin. Gastrin antisense-transfected cell lines have previously been shown to have reduced growth *in vitro* and are poorly tumorigenic *in vivo* (Singh *et al*, 1996; Harris *et al*, 2004b; Smith *et al*, 2004). However, siRNAs potentially provide a more powerful and specific mechanism for the downregulation of genes than traditional antisense approaches (Aoki *et al*, 2003) and have stringent sequence specificity (Elbashir *et al*, 2001a). The data described in this paper demonstrate a protective role for endogenous gastrin in a range of GI cancer cells using an siRNA specific for gastrin.

## MATERIALS AND METHODS

### Cell culture

PAN-1 is a human pancreatic cell line derived from a poorly differentiated human pancreatic adenocarcinoma within the Academic Unit of Cancer Studies, University of Nottingham, UK. HCT116, a poorly differentiated human colon cell line, was obtained from ECACC (ref. no. 91091005). C170HM2 is a human colorectal tumour cell line originally derived from a poorly differentiated tumour (Watson *et al*, 1993). MGLVA1 is an ascitic variant of the gastric cell line, MKN 45G (Watson *et al*, 1990).

All cell lines were routinely cultured in RPMI 1640 culture medium (Gibco, Paisley, UK) containing 10% (v/v) heat-inactivated fetal bovine serum (Sigma, Poole, UK) at 37°C in 5% CO_2_ and humidified conditions.

### Small interfering RNA synthesis

Target sites within the gastrin gene for siRNA synthesis were chosen for the following properties (Elbashir *et al*, 2001b):

- preceded by AA in the gene sequence
- avoidance of the extreme 5′ or 3′ ends of the gene
- 30–60% GC content
- absence of long stretches of A's
- lack of homology with other genes using BLAST

Their sequences are given in Table 1. Initial experiments were carried out with siRNAs synthesised *in vitro* using the Silencer siRNA Construction Kit (Ambion, Austin, TX, USA), according to the manufacturer's instructions. Briefly, template DNA oligonucleotides containing the sense or antisense of the target sequence, followed by the leader sequence CCTGTCTC at the 3′ end, were synthesised commercially (Helena Bioscience, Gateshead, UK). The T7 promoter-containing primer was annealed to the template oligonucleotides and dsDNA created using Klenow DNA polymerase. The sense and antisense dsDNAs were used as templates to synthesise the antisense or sense strands of RNA, respectively, using T7 RNA polymerase. The two RNA strands were annealed together, digested with DNase and RNase to remove the template DNA and leader sequence, then column-purified and stored at −80°C. The concentration of the siRNAs was calculated by measuring their optical density (OD) at 260 nm.

Small interfering RNAs were also synthesised commercially as separate strands that were annealed and PAGE-purified (Dharmacon, Lafayette, CO, USA). A fluorescent version of tg8 was also synthesised with a TAMRA label at the 5′ end of the sense strand.

### Small interfering RNA transfection

Cells (5 × 10^4^) were plated out in 24-well plates and cultured overnight in normal growth medium. In initial experiments, two transfection reagents, siPortAmine and siPortLipid (Ambion), were tested under a range of conditions to optimise the protocol. The following protocol was used to generate the data described in the paper. Each well was transfected using 4 *μ*l of siPortAmine and 1 *μ*l of siRNA, to give final concentrations of 25 nM of *in vitro*-synthesised siRNAs or up to 0.5 *μ*g ml^−1^ of commercially synthesised siRNA. The siPortAmine was vortexed, added drop by drop to 45 *μ*l Opti-MEM1 (Invitrogen, Paisley, UK), vortexed and incubated for 30 min at room temperature. The diluted transfection reagent was added to the siRNA, mixed by pipetting and incubated for a further 30 min. The growth medium was removed from the cells and replaced by 200 *μ*l Opti-MEM1 and then overlaid with the transfection reagent : siRNA complex, with rocking. The plates were incubated for 6 h at 37°C, 5% CO_2_, after which 1 ml growth medium was added and the plates were returned to the incubator.

### Real-time PCR

RNA was extracted using RNABee (Biogenesis, Poole, UK) and complementary DNA (cDNA) synthesised using Superscript II and random hexamers as described previously (McWilliams *et al*, 1998). For each RNA, a cDNA-negative control (no RT or primer) was also synthesised. PCR for gastrin and the housekeeping gene was routinely carried out using 5 *μ*l of 1 : 5 cDNA and 20 *μ*l of reaction mix consisting of 1 × reaction buffer, 1 : 2000 SybrGreen, MgCl_2_, dNTP mix, 1 *μ*l of primer and Hot GoldStar Taq (Eurogentec, Romsey, UK). The sequences of the primers used are shown in Table 2. In addition, oligoadenylate synthase 1 (OAS), signal transducer and activator of transcription 1 (STAT1) and hypoxanthine guanine phosphoribosyl transferase (HPRT) genes were detected with Taqman primer sets (Applied Biosystems, Warrington, UK), using the same reaction mix as above but without SybrGreen.

Complementary DNAs were tested in triplicate and, in addition, cDNA-negative and ‘no template’ controls were analysed to ensure that the signal generated was derived from RNA and not from genomic DNA, primer-dimers or any of the cDNA or PCR reagents. The samples were run on a GeneAmp 5700 Sequence Detector Real-Time PCR machine (Applied Biosystems) using the following programme: 50°C for 2 min, 95°C for 10 min, 40 cycles of 95°C for 15 s and 60°C for 1 min with products being detected using SYBR Green or the fluorescent Taqman probe. The number of cycles required to reach a fluorescence threshold (*C*_t_) during the exponential phase of amplification was determined for the test gene and for the housekeeping gene HPRT. Results are usually presented as relative gene expression in comparison to HPRT and a reference treatment using the 2^−ΔΔ*C*t^ method (Livak and Schmittgen, 2001), where 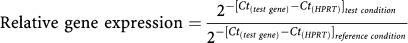


To demonstrate differences in expression levels between different cell lines, data are shown using the 2^−Δ*C*t^ equation where 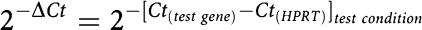 95% confidence intervals for the mean gastrin expression were calculated based on the variation between the replicates for each sample and are indicated on each graph as error bars.

### Green fluorescent protein-tagged gastrin

The complete gastrin coding sequence was amplified by PCR and cloned upstream of the green fluorescent protein (GFP) coding sequence in the plasmid pHRGFP-C (Stratagene, LaJolla, CA, USA), under the control of a CMV promoter, using the *Bam*H1 and *Xho*I restriction sites to create a plasmid pGasGFP. A complete Kozak sequence was incorporated into the forward primer, involving modification of the fourth nucleotide of the gastrin coding sequence from C to G (indicated in the primer sequence by underlining). The primers used were as follows: Forward: CGCGGATCCGCCGCCGCCATGGAGCGACTGTG TGTG Reverse: CCGCCGCTCGAGGCCGAAGTCCATCCATC

The unmodified pHRGFP-C plasmid was used as a vector control. As it lacks a Kozak sequence, no GFP expression was expected.

Dual transfection of plasmids and siRNAs was carried out by preparing the siRNA transfection mix as above but in half the volume, and preincubating 500 ng of plasmid with 1 *μ*l Lipofectamine (Invitrogen, Paisley, UK) in 25 *μ*l serum-free medium. The two transfection reagents were then mixed immediately before addition to the cells.

### Antibody staining of cytospins

Following transfection, cells were stored, fixed in 4% formalin overnight at 4°C, then washed twice in PBS after each of the following steps. Cytospins were made in a Shandon cytospin 4 using 10^4^ cells per slide. Cells were permeabilised by incubation with PBS containing 0.5% Triton X-100 for 5 min at 4°C. Nonspecific binding sites were blocked by a 30 min incubation at room temperature in PBS containing 3% BSA and 1% glycine followed by PBS containing 10% normal swine serum (NSS). The primary antibody (rabbit anti-progastrin antibody, raised against amino acids 6–14 of progastrin conjugated to diphtheria toxin (DT) and purified against the BSA peptide conjugate; (Aphton Corp., Philadelphia, PA, USA) or control antibody (rabbit anti-DT) was added at a final concentration of 2.5 *μ*g ml^−1^ in PBS containing 1% NSS and incubated overnight at 4°C. Binding of the antibody was detected using FITC-conjugated swine anti-rabbit immunoglobulin at a dilution of 1 : 40 (Dako, Ely, UK) and the cells were counterstained with Hoechst dye before examination under a fluorescent microscope.

### Growth assays

Cell growth was measured using a standard methyl thiazoyl tetrazolium (MTT) assay (Watson *et al*, 1996). Briefly, following transfection with siRNAs, cells were grown overnight, then trypsinised, plated out into 96-well plates as replicates and incubated overnight in growth medium. The following day, the medium was replaced by RPMI 1640 culture medium supplemented with 1% serum or serum-free OptiMem1, and the cells were grown for a further 96 h. In some experiments, OptiMem1 was supplemented with 10 nM amidated gastrin (G17, Aphton, USA), 10 nM glycine-extended gastrin (Gly-G17, Aphton, USA) or the CCK2-R inhibitor, YM022 (James Black Foundation, Dulwich, UK) at 500 nM. At intervals following transfection, the medium was replaced by fresh medium containing MTT at 1 mg ml^−1^ and incubated for 4 h. The medium was removed, the incorporated MTT dissolved in DMSO and the OD at 550 nm was read.

### Caspase 3 inhibitor assay

Cells undergoing apoptosis were identified by addition of a fluorescent caspase 3 inhibitor, FITC-DEVD-FMK (Calbiochem, Nottingham, UK), according to the manufacturer's instructions. Cells were harvested using trypsin–EDTA, resuspended in fresh medium and incubated with the inhibitor at the recommended final working concentration for 1 h at 37°C. The cells were washed twice in the wash buffer provided, fixed in formalin and analysed on a Beckman-Coulter XL-MCL flow cytometer. The percentage of apoptotic cells was calculated, using untreated cells to define the baseline.

### Statistical analysis

The significance of differences in gene expressing between cell lines was calculated using ANOVA with the Bonferroni multiple comparison test. The significance of differences between growth and rates of apoptosis in cells transfected with the gastrin siRNA and control siRNA was measured using the Student's *t*-test. Differences were considered to be significant if *P*<0.05.

## RESULTS

### Localisation of suitable targets within gastrin

**Figure 1:**
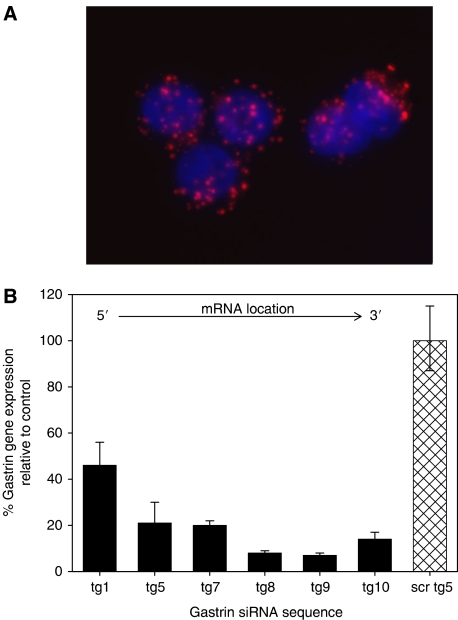
(**A**) siRNA transfection efficiency. PAN-1 cells transfected with a TAMRA-labelled gastrin siRNA 24 h post-transfection (× 64 magnification). The siRNA is associated with vesicle-like structures in the cytoplasm of the cells. (**B**) Expression of the gastrin gene in cells transfected with different gastrin siRNAs. PAN-1 cells were transfected with six different gastrin siRNAs and gastrin expression was measured by real-time PCR. Expression relative to cells transfected with the scrambled control siRNA (scrtg5) and the housekeeping gene, HPRT, is shown. Error bars indicate the 95% confidence intervals. The relative position of each siRNA within the gastrin sequence is indicated.

A fluorescent TAMRA-labelled siRNA (tg8) was used to measure the efficiency of transfection. At 24 h after transfection, almost 100% of the cells were fluorescent with the signal associated with vesicle-like structures in the cytoplasm of the cells (Figure 1A). Small interfering RNAs directed against six different targets within gastrin and a control siRNA consisting of a scrambled version of the tg5 sequence were then used to transfect PAN-1 cells. Gene expression was measured 24 h after transfection by real-time PCR. Use of the most effective siRNAs, tg8 and tg9, resulted in a 93% decrease in gastrin gene expression relative to that in cells treated with the control siRNA and the housekeeping gene HPRT. The remaining siRNAs gave between 64 and 86% downregulation of the gastrin gene (Figure 1B). Tg8 was used for the remainder of the studies and synthesised commercially together with a scrambled control.

### The gastrin siRNA is effective in a range of cell lines with different gastrin expression levels and is able to overcome transcriptional upregulation of the gastrin gene by EGF

**Figure 2:**
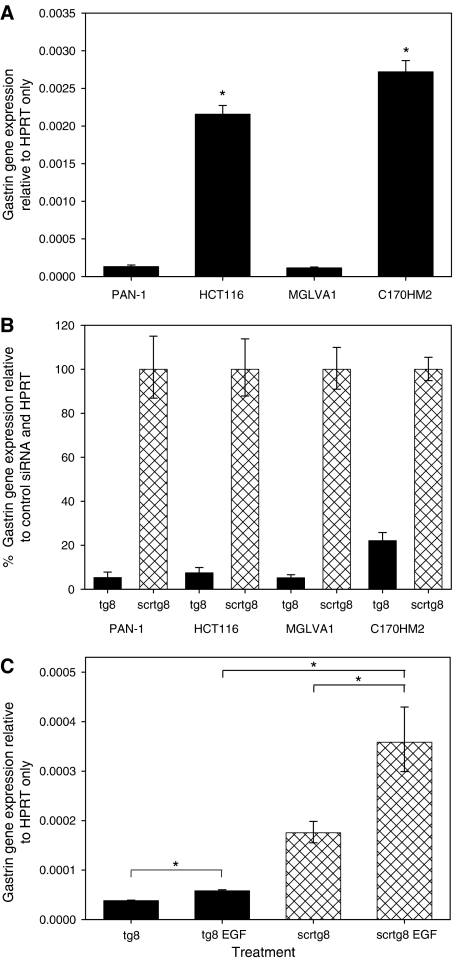
Effectiveness of gastrin siRNA in a range of GI cancer cell lines. (**A**) Relative expression of gastrin in four cell lines (PAN-1, HCT116, MGLVA1 and C170HM2) is shown using the 2^−Δ*C*t^ formula. A significant difference in expression compared with that in PAN-1 (*P*<0.001) is indicated by ^*^. (**B**) The downregulation of gastrin gene expression in cells treated with the tg8 gastrin siRNA is shown relative to cells treated with the control scrtg8 siRNA using the 2^−ΔΔCt^ formula. (**C**) Gene expression in PAN-1 cells on d2 after transfection with gastrin (tg8) or control (scrtg8) siRNA and 24 h treatment with or without 10 *μ*g ml^−1^ EGF. Data are expressed relative to the housekeeping gene, HPRT. Significant differences (*P*<0.001) are indicated by ^*^. Error bars indicate the 95% confidence intervals.

The tg8 and scrtg8 siRNAs were used to transfect three additional cell lines, HCT116 (colorectal), C170HM2 (liver-metastasising variant of a colorectal cell line) and MGLVA1 (gastric). Basal gastrin gene expression in the four cell lines is shown in Figure 2A. Expression in HCT116 and C170HM2 cells was significantly higher (approximately 15 × and 20 × , respectively; *P*<0.001 for both) than that in the PAN-1 and MGLVA1 cells. Nonetheless, in each of the cell lines, between 80 and 95% downregulation of the gastrin gene was achieved in cells treated with gastrin siRNA (Figure 2B). In addition, the ability of the gastrin siRNA to overcome upregulation of the gastrin gene by EGF was demonstrated in PAN-1 cells (Figure 2C).

### Downregulation of the gastrin gene by the siRNA is dose-dependent and is maintained *in vitro* for more than 1 week

**Figure 3:**
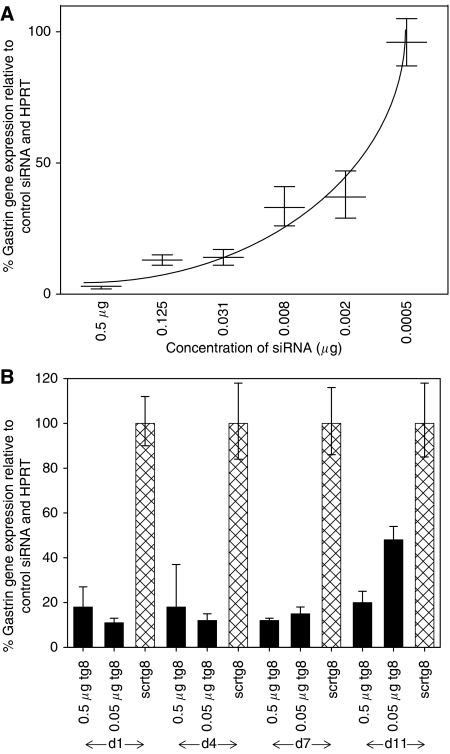
Concentration-dependent effects of the gastrin siRNA and longevity of downregulation of the gastrin gene. (**A**) Gastrin gene expression in HCT116 cells transfected with a range of concentrations of gastrin (tg8) siRNA, expressed relative to the housekeeping gene, HPRT, and cells transfected with the same concentration of the control (scrtg8) siRNA. Error bars indicate the 95% confidence intervals. (**B**) Gene expression in PAN-1 cells at d1, d4, d7 or d11 after transfection with 0.5 or 0.05 *μ*g gastrin (tg8) or control (scrtg8) siRNA. Data are expressed relative to the control siRNA and the housekeeping gene, HPRT. Error bars indicate the 95% confidence intervals.

The tg8 and scrtg8 siRNAs were tested at a range of concentrations against HCT116 cells, one of the cell lines expressing the highest level of gastrin RNA. Downregulation of the gastrin gene was dose-dependent (Figure 3), with 60% downregulation of the gene still achieved at the lowest effective dose of siRNA (0.02 *μ*g).

**Figure 4:**
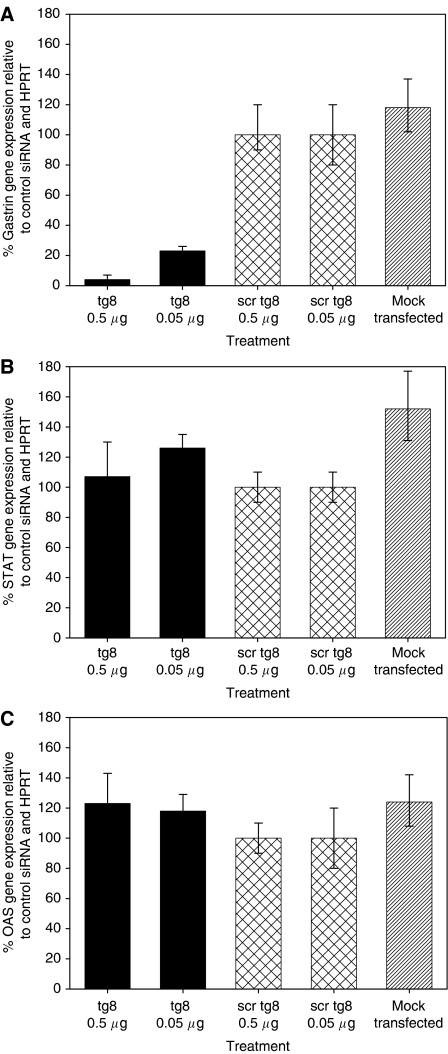
Absence of induction of the innate interferon response by gastrin siRNA. Gastrin, OAS and STAT1 gene expression in PAN-1 cells treated with gastrin (tg8), control (scrtg8) siRNA or no siRNA (mock transfected). Error bars indicate the 95% confidence intervals.

To investigate the duration of the effectiveness of the siRNA, PAN-1 cells were transfected with 0.5 or 0.05 *μ*g tg8 or scrtg8 siRNA, harvested at a range of time intervals following transfection and analysed for gene expression by real-time PCR. The effect of the tg8 siRNA was prolonged. At the highest concentration of siRNA used, 90% downregulation of the gene was maintained up to 7 days after transfection, dropping to 80% at day (d) 11. At the lower concentration of siRNA, 85% downregulation was maintained up to d7, but dropped to 53% by d11 (Figure 4). When a similar experiment (data not shown) was carried out with HCT116 cells, which have a higher basal level of gastrin expression, 60% downregulation of the gene was maintained up to d8 after transfection, provided that the higher concentration of siRNA was used. Thus, for the remainder of experiments, cells were transfected with 0.5 *μ*g siRNA per 5 × 10^4^ cells.

### The innate interferon response is not induced by the gastrin siRNA

To investigate whether transfection with gastrin siRNA results in induction of the innate interferon response, expression of STAT1 and OAS was measured in HCT116 cells transfected with tg8 or scrtg8 siRNA or mock-transfected cells treated with the transfection reagent but without siRNA. Whereas there was a significant difference comparing gastrin expression (Figure 4A) in tg8-treated cells with scrtg8- or mock-treated cells (*P*=0.005), there was no significant difference in STAT1 (Figure 4B) or OAS (Figure 4C) expression (*P*>0.28), suggesting that the innate interferon response is not induced by these siRNAs under these conditions.

### Downregulation of GFP-tagged and endogenous gastrin

**Figure 5:**
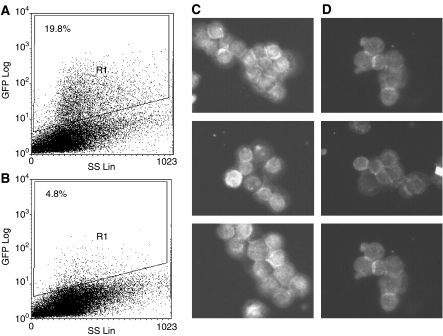
Downregulation of GFP-tagged and endogenous gastrin protein expression by gastrin siRNA. Cells transfected with (**A**) a GFP-tagged gastrin plasmid and treated with control scrtg8 siRNA or (**B**) gastrin tg8 siRNA are shown 24 h after transfection. The percentage of positive cells is indicated and there was a significant difference (*P*<0.0001) between the two treatments. Cells transfected with (**C**) scrtg8 or (**D**) gastrin tg8 siRNA were immunostained for endogenous gastrin expression using an anti-C-terminal antibody 72 h after transfection. Three fields are shown for each treatment.

Owing to the complexity of the products arising from the gastrin gene and the low levels of gastrin protein produced by GI tumour cells, the effect of siRNA transfection on gastrin protein expression was initially analysed using HCT116 cells transfected with a GFP-tagged gastrin gene. Four replicates of each cotransfection were carried out. One replicate for each treatment is shown in Figure 5A and B. The average percentage of fluorescent cells following transfection with pGasGFP and the control siRNA was 19.8, whereas only 4.8% of cells cotransfected with pGasGFP and the gastrin siRNA were positive. Thus, there was a significant reduction in the proportion of GFP-tagged gastrin-expressing cells within 24 h of treatment with the gastrin siRNA (*P*<0.00001). However, a different pattern was seen when endogenous gastrin expression in siRNA-treated HCT116 cells was measured using a monoclonal antibody to amino acids 6–14 of progastrin. There was no apparent difference between tg8-and scrtg8-treated cells at early time points with the first detectable reduction in progastrin staining observed at d3 after transfection (Figure 5C and D), when there was still some residual staining of tg8-treated cells.

### Endogenous gastrin enhances survival of serum-starved GI cancer cells

**Figure 6:**
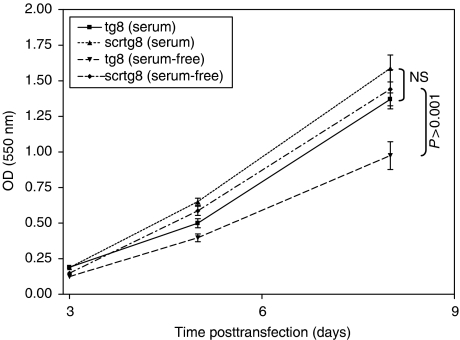
Survival of gastrin siRNA-transfected cells in the presence and absence of serum. Growth of PAN-1 cells transfected with gastrin tg8 siRNA or the control scrtg8 siRNA in serum containing 10% or serum-free medium over 8 days following transfection. At d3 and d6, there was significantly lower survival in the tg8- treated cells than the scrtg8-treated cells (*P*=0.004 and 0.001 for serum and no serum, respectively, at d3, and *P*=0.06 and 0.02, respectively, at d6).

To measure the role of endogenous gastrin in cell survival following serum starvation, PAN-1 were cultured in medium supplemented with serum or serum-free medium (Figure 6A). Cell survival was assessed at a range of time points using an MTT assay. Under both sets of conditions, growth was lower in the gastrin siRNA-treated cells than in cells treated with the control siRNA. However, the effects reached significance only under serum-free conditions (*P*<0.001), with 32% inhibition in serum-free medium compared with 13% (NS) in medium supplemented with serum at d8. Consistent with the delay in downregulation of endogenous gastrin protein expression following siRNA treatment, clear differences in cell survival were not apparent until approximately d6 following transfection.

**Figure 7:**
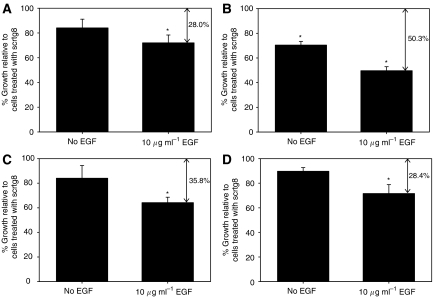
Survival of gastrin siRNA-transfected cancer cells. Survival of (**A**) PAN-1, (**B**) C170HM2, (**C**) HCT116 and (**D**) MGLVA1 cells in serum-free medium with or without the addition of 10 *μ*g ml^−1^ EGF following transfection with gastrin (tg8) or control (scrtg8) siRNAs. Growth of tg8-transfected cells is shown as a percentage of scrtg8-treated cells. Significant differences (*P*<0.05) are indicated by ^*^.

As the gastrin siRNA reduces induction of the gastrin gene expression by EGF, cell survival was measured for each cell line in the presence or absence of 10 *μ*g ml^−1^ EGF on d6 after transfection. The experiment was carried out on a minimum of three separate occasions and representative data for a single experiment for each cell line are shown in Figure 7. Although there was some variation between experiments, in all four cell lines there was reduced cell survival in the tg8-compared with the scrtg8-treated cells. Reduced thymidine incorporation was also observed in PAN-1 cells over a similar time frame (data not shown).

The effects were most marked in the C170HM2 cells, which expressed the highest level of gastrin. In the absence of EGF, there was a significant reduction in cell survival on each of the three occasions they were tested (% cell survival of 63.1, 75.0 and 68.5), whereas in the remaining three cell lines, in the absence of EGF, significance was reached in only a proportion of separate experiments. Similar reductions in growth were seen in PAN-1 and C170HM2 cells treated with two other gastrin siRNAs, tg5 and tg7 (data not shown), suggesting that this effect is specifically related to downregulation of the gastrin gene. In cells treated with EGF, a significant difference in growth was seen in every independent experiment and the percentage reduction in growth was greater.

### Downregulation of endogenous gastrin leads to increased apoptosis in GI cancer cells exposed to serum-free conditions

**Figure 8:**
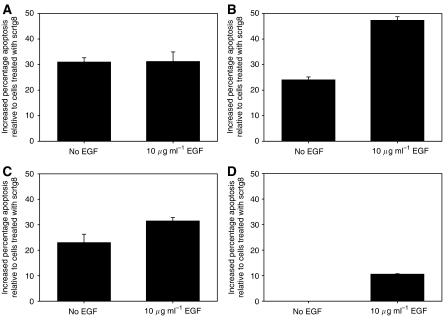
Apoptosis in gastrin siRNA-transfected cancer cells. Proportion of apoptotic cells at d4 following treatment with gastrin (tg8) or the control (scrtg8) siRNA in the presence or absence of 10 *μ*g ml^−1^ EGF. Apoptosis is shown relative to scrtg8-treated cells. Significant differences (*P*<0.05) are indicated by ^*^. (**A**) PAN-1, (**B**) C170HM2, (**C**) HCT116 and (**D**) MGLVA1.

As gastrin is thought to be anti-apoptotic as well as acting as a growth factor, the effect of downregulation of endogenous gastrin on apoptosis in the four cell lines following exposure to serum-free conditions was also investigated. A fluorescent caspase 3 inhibitor was used to identify cells undergoing apoptosis, and the proportion of apoptotic cells, following transfection with the gastrin or control siRNA, was measured. Basal levels of apoptosis in the cell lines in serum-free medium varied between the different cells and in different experiments but was in the range of 10–30%. The data at d4 following transfection are shown in Figure 8. There was increased apoptosis in the cells treated with the gastrin siRNA compared with the control siRNA, except in the MGLVA1 cells. In the absence of EGF, the difference was significant in the PAN-1 and C170HM2 cells. The effect was enhanced in the presence of EGF, with a significant difference seen additionally in the HCT116 cells. Significance was not reached in the MGLVA1 cells under either condition.

### Amidated gastrin and glycine-extended gastrin enhance growth of gastrin siRNA-treated cells

**Figure 9:**
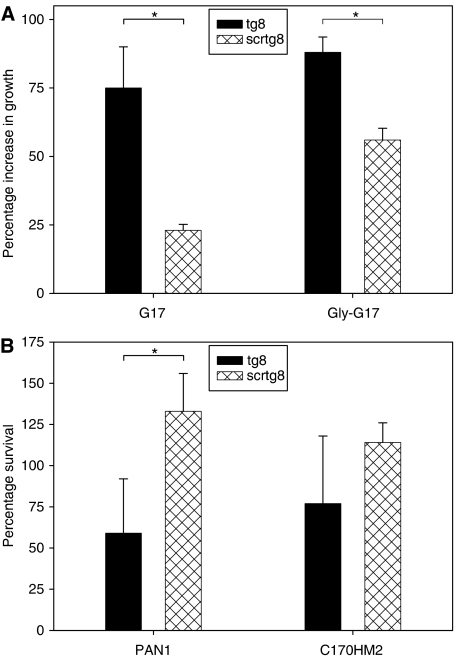
Growth of siRNA-transfected cells in the presence of gastrin peptides and a CCK2-R antagonist, (**A**) the growth of gastrin (tg8) or control (scrtg8) siRNA-transfected C170HM2 cells treated with 10 nM amidated (G17) or glycine-extended (Gly-G17) gastrin. The percentage increase in growth compared with transfected cells alone is indicated. There was a significant increase in the growth of peptide-treated gastrin siRNA-transfected cells compared with control siRNA-transfected cells (*P*<0.001 for both G17 and GlyG17, indicated by ^*^). (**B**) The growth of gastrin (tg8) or control (scrtg8) siRNA-transfected Pan1 and C170HM2 cells treated with 500 nM YM022 is shown. There was a significant further reduction in cell survival in tg8-transfected Pan1 cells following treatment with YM022 (41%, *P*=0.003) is shown. There was a 23% reduction in cell survival in C170HM2 cells but this did not reach significance.

To investigate the ability of exogenous gastrin to restore growth of cells treated with the gastrin siRNA, siRNA-transfected cells were treated with either amidated (G17) or glycine-extended gastrin (Gly-G17) at 10 nM. The growth of control siRNA-treated C170HM2s was increased by 23 and 56%, following treatment with G17 and Gly-G17 respectively, and growth of the control siRNA-treated cells was increased by 75 and 88%, respectively (Figure 9A). There was a significantly greater increase in growth in gastrin siRNA-transfected cells treated with G17 and GlyG17 than in control siRNA-treated cells (*P*<0.001 for both).

### Treatment of gastrin siRNA-treated cells with YM022 further reduced the survival of gastrin siRNA-transfected cells

The effect of the CCK2-R antagonist YM022 on survival of the gastrin siRNA-transfected cells was investigated in PAN-1 and C170HM2 cells. The antagonist had no significant effect on the growth of the control siRNA-treated cells but significantly reduced the survival of gastrin siRNA-transfected PAN-1 cells by 41% (Figure 9B, *P*=0.003). Survival of gastrin siRNA-transfected C170HM2 cells was reduced by 23% following treatment with YM022, but this did not reach significance.

## DISCUSSION

Gastrin is involved in the establishment of a range of GI tumours. Here, using an siRNA that specifically targets gastrin, we show that endogenous gastrin plays a role in the survival of cell lines representing gastric, colorectal and pancreatic adenocarcinoma. Downregulation of gastrin resulted in reduced survival of cells exposed to serum-free conditions and was attributable at least in part to an increase in the rate of apoptosis in the gastrin siRNA-treated cells.

Initially, a number of siRNAs directed against potential targets within the gastrin gene were investigated for their effectiveness at mediating downregulation of the gastrin mRNA transcript. Interestingly, the most effective siRNAs were clustered towards the 3′ end of the transcript. This may be a result of easier access of the siRNA to its target due to reduced structural complexity in this region of the RNA as previously described (Luo and Chang, 2004; Yoshinari *et al*, 2004). The effects of the gastrin siRNA appear to be specific. The possibility of off-target effects (Scacheri *et al*, 2004) and induction of the interferon response (Sledz *et al*, 2003) has been suggested in the literature. A BLAST search was included in the design of the siRNAs used in this study and no matches to other genes were identified. Similar growth effects were seen with two additional gastrin siRNAs, and in all experiments a scrambled siRNA was used as a control for potential nonspecific effects due to the addition of siRNAs to the cell or transfection. In addition, we saw no induction of either OAS or STAT1 genes in response to the gastrin or control siRNA and the delay in the effects of the siRNA also argues against the involvement of the interferon response in the growth effects observed as this innate response is generally induced rapidly.

We were able to demonstrate rapid downregulation of plasmid-encoded GFP-tagged gastrin peptides at the protein level within 24 h of treatment with the gastrin siRNA. However, there was an apparent delay of 2–3 days in downregulation of endogenous gastrin protein. Similar effects have been described previously for a number of genes (Wu *et al*, 2004) and may relate to slow rates of turnover of individual proteins within the cell or mechanisms that allow continued expression of protein from the small residual pool of mRNA; such mechanisms may not be active on recombinant vector-derived protein.

This apparent delay in downregulation of endogenous gastrin protein correlated with the time frame for measurement of the biological effects of downregulation of gastrin by the siRNA, which were delayed to approximately d5 following transfection. Downregulation of gastrin in the presence of serum resulted in a statistically significant reduction in cell survival, but the effect was small. However, in serum-free conditions, downregulation of gastrin reduced cell survival by up to 50% in the presence of EGF, the effect being most marked in the colorectal cell lines, especially C170HM2. This cell line has the highest constitutive levels of gastrin expression and may, therefore, be more dependent on gastrin for maintenance of the cell cycle.

The reduced survival of cells in which endogenous gastrin has been knocked down is in part due to an increase in apoptosis in these cells. The effects on apoptosis parallel the results of the cell survival assays, with the strongest effects seen in the colorectal cell lines, especially C170HM2, and with no significant effects on apoptosis seen in MGLVA1. A raised level of caspase 3 in cells treated with gastrin antisense and exposed to camptothecin has been reported previously in HCT116 cells (Wu *et al*, 2000).

Both gastrin and control siRNA-treated cells were responsive to amidated and glycine-extended gastrin. Interestingly, the gastrin siRNA-treated cells were more responsive, with not only greater percentage increase but also a higher final cell number than the control siRNA-treated cells treated with the peptides. This result further suggests that downregulation of the gastrin gene may lead to increased expression of the receptors for these peptides.

This hypothesis is also supported by the data from the gastrin siRNA-transfected cells treated with the CCK2-R antagonist, YM022. The control siRNA-treated cells were unresponsive to YM022, suggesting that CCK2-R is not involved in the basal growth of these cells. This is in keeping with our findings that classical CCK2-R expression is undetectable in these cell lines by real-time PCR (data not shown) or using a nested PCR method that avoids potential artefacts associated with amplification of genomic DNA (Grabowska *et al*, 2004). It suggests that the gastrin-dependent cell survival, which is overcome by the gastrin siRNA, is not effected through the classical CCK2-R.

However, there was a decrease in the survival of the gastrin siRNA-treated cells in response to YM022. This could be explained by upregulation of one or more gastrin receptors that are inhibited by YM022, with either constitutive activity or with stimulation by residual endogenous gastrin. Thus, the percentage reduction in cell growth resulting from treatment with gastrin siRNA may be an underestimate of the role of gastrin in the basal growth of these cells if downregulation of gastrin leads to upregulation of alternative pathways that enhance cell growth.

The ability of the gastrin siRNAs to reduce survival of these GI cell lines is further evidence of the importance of autocrine and/or intracrine gastrin loops in the growth of these GI cancer cells. The effect of the gastrin siRNA is of a similar order of magnitude to that which has been described before when antibodies to gastrin peptides, receptor antagonists or antisense have been used to downregulate gastrin (Hoosein *et al*, 1988; Singh *et al*, 1996; Smith *et al*, 1996, 2004), and suggests that gastrin is only one of a number of factors contributing to survival of GI cancer cells. However, these data may also underestimate the role of endogenous gastrin. In the case of antibodies or receptor antagonists, they are effective against only a subset of gastrin isoforms. The siRNA described here is able to effectively downregulate gastrin at the RNA level and so should affect all products of the gastrin gene, but as downregulation of the protein is delayed and incomplete, there is only a narrow window in which the effect of gastrin knockdown can be measured. Further investigation of the mechanisms underlying gastrin translation and protein turnover would be beneficial. In addition, future studies will require systems to deliver the siRNA over a longer time frame, possibly through the use of plasmid-encoded small hairpin siRNAs or development of alternative approaches such as microRNAs that inhibit translation of the gastrin transcript. This will provide a model that will allow a more detailed study of the complex interactions between the different isoforms of gastrin and gastrin receptors expressed by these cells.

## Figures and Tables

**Table 1:** Gastrin siRNA target sequences

**Target**	**Target sequence**	**% GC**	**Position in gene**
tg1	AAGCTTCTTGGAAGCCCCGCT	57.1	59
tg5	AAGAAGCAGGGACCATGGCTG	57.1	220
tg7	AAGAAGAAGAAGCCTATGGAT	38.1	245
tg8	AAGAAGAAGCCTATGGATGGA	42.9	248
tg9	AAGAAGCCTATGGATGGATGG	47.6	251
tg10	AAGCCTATGGATGGATGGACT	47.6	254
scr tg5	AAGAGATGTAAGGCCAGGCCG	57.1	NA
scr tg8	AAGCGAAGAAACGAGGTGTAT	42.9	NA

NA: not applicable.

**Table 2:** Primers used for real-time PCR

**Primer**	**Primer sequence (5′ → 3′)**
Gastrin F	CCACACCTCGTGGCAGAC
Gastrin R	TCCATCCATCCATAGGCTTC
HPRT F	GACCAGTCAACAGGGGACAT
HPRT R	CGACCTTGACCATCTTTGGA
